# Emergency department presentations with suicide and self-harm ideation: a missed opportunity for intervention?

**DOI:** 10.1017/S2045796023000203

**Published:** 2023-04-18

**Authors:** E. Ross, S. Murphy, D. O’Hagan, A. Maguire, D. O’Reilly

**Affiliations:** 1 Centre for Public Health, Queen’s University Belfast, Belfast, Northern Ireland; 2 Service Development, Screening and Health Improvement, Public Health Agency, Ballymena, Northern Ireland

**Keywords:** alcohol abuse, epidemiology, mental health, suicide, suicide ideation

## Abstract

**Aims:**

Suicidal ideation constitutes a central element of most theories of suicide and is the defining facet separating suicide from other causes of death such as accidents. However, despite a high worldwide prevalence, most research has focused on suicidal behaviours, such as completed suicide and suicide attempts, while the greater proportion who experienced ideation, which frequently precedes suicidal behaviour, have received much less attention. This study aims to examine the characteristics of those presenting to EDs with suicidal ideation and quantify the associated risk of suicide and other causes of death.

**Methods:**

Retrospective cohort study was performed based on population-wide health administration data linked to data from the Northern Ireland Registry of Self-Harm and centrally held mortality records from April 2012 to December 2019. Mortality data, coded as suicide, all-external causes and all-cause mortality were analysed using Cox proportional hazards. Additional cause-specific analyses included accidental deaths, deaths from natural causes and drug and alcohol-related causes.

**Results:**

There were 1,662,118 individuals aged over 10 years, of whom 15,267 presented to the ED with ideation during the study period. Individuals with ideation had a 10-fold increased risk of death from suicide (hazard ratio [HR_adj_] = 10.84, 95% confidence interval [CI] 9.18, 12.80) and from all-external causes (HR_adj_ = 10.65, 95% CI 9.66, 11.74) and a threefold risk of death from all-causes (HR_adj_ = 3.01, 95% CI 2.84, 3.20). Further cause-specific analyses indicated that risk of accidental death (HR_adj_ = 8.24, 95% CI 6.29, 10.81), drug-related (HR_adj_ = 15.17, 95% CI 11.36, 20.26) and alcohol-related (HR_adj_ = 10.57, 95% CI 9.07, 12.31) has also significantly increased. There were few socio-demographic and economic characteristics that would identify which of these patients are most at risk of suicide or other causes of death.

**Conclusions:**

Identifying people with suicidal ideation is recognized to be both important but difficult in practice; this study shows that presentations to EDs with self-harm or suicide ideation represent an important potential intervention point for this hard-to-reach vulnerable population. However, and unlike individuals presenting with self-harm, clinical guidelines for the management and recommended best practice and care of these individuals are lacking. Whilst suicide prevention may be the primary focus of interventions aimed at those experiencing self-harm and suicide ideation, death from other preventable causes, especially substance misuse, should also be a cause of concern.

## Introduction

Suicidal ideation (SI) is the defining facet separating suicide from other causes of death such as accidents. It constitutes a central element of most theories of suicide, with a separation of the factors leading to SI from those promoting or presaging the transition from ideation to suicidal actions (Klonsky *et al*., 2016). It has been estimated that 9% of the population across the world will experience SI at some point in their lives, 30% of whom will go on to make a suicide attempt (Nock *et al*., 2008).

However, research has focused on suicidal behaviours, such as completed suicide and suicide attempts and acts of self-harm (SH), while the greater proportion who suffer from ideation have received much less attention, perhaps because it is an elusive, ephemeral and often fluid construct (Jobes and Joiner, 2019). It is further complicated by the absence of a universally accepted definition and the breadth of the phenomenon, spanning from a general tiredness of life and desire for death through to active suicide planning, from fleeting thoughts to incessant and disturbing ruminations. Consequently, estimates of both prevalence and the associated outcomes makes comparisons across studies difficult (Berman, 2018; Goodfellow *et al*., 2019; Harmer *et al*., 2023). A recent meta-analysis found an overall threefold increased risk of suicide (McHugh *et al*., 2019) though noted very high and unexplained levels of between-study heterogeneity which was attributed to differences in methodological design, with studies utilizing structured methods producing weaker associations than those using clinically derived indicators. Furthermore, many studies were based on small study sizes, making robust estimation of the associated risk challenging, or on high-risk groups, making it difficult to generalize to the general population (McHugh *et al*., 2019).

However, most patients with SI do not take their lives, and it is notoriously difficult to predict who is most at risk of doing so (Chan *et al*., 2016; Steeg *et al*., 2018). The high healthcare usage in the period preceding suicide or suicide attempts (Stene-Larsen and Reneflot, 2019) suggests that healthcare professionals have potential opportunity to identify and intervene in this pathway. However, to date, and with the notable exception of one study (Goldman-Mellor *et al*., 2019), there has been little study of patients with SI who present to ED. This is in stark contrast to SH patients for whom recommendation for best practice and care are now included in the National Institute for Health and Care Excellence (NICE) clinical guidelines in England (NICE, 2004; NICE, 2022).

Northern Ireland (NI) has a high prevalence of mental ill-health and the highest rate of SH in the United Kingdom (PHA, 2019) and is unique as it holds the only national registry of individuals with suicidal or SH ideation presenting to emergency departments (EDs). This provides an unparalleled opportunity to explore the association between ideation and subsequent suicide on a population-wide level (Carr *et al*., 2016). Previous analysis of the Northern Ireland Registry of Self-Harm (NISHR) shows that the incidence rate for ideation is about half that for SH, and although they share similar age profiles, rates of ideation presentation are higher for males than females (Griffin *et al*., 2019). Repeat presentations to the ED with ideation are high, and about one in five ideators will subsequently present with SH over a 5-year period (Griffin *et al*., 2020). However, it has not been previously possible to quantify the risk of suicide and other causes of death following presentation to ED with ideation.

The aims of this study were to describe the characteristics of individuals who present with suicidal or SH ideation; to quantify the risk of suicide and other causes of death and to identify, within those presenting with ideation, the factors associated with increased mortality risk. Although suicide is likely to represent many excess deaths in those presenting with ideation, we included additional analyses to include drug and alcohol-related deaths, accidental deaths and deaths from external causes and all-cause mortality.

## Methods

### Data sources and measures

Data on ideation presentations are extracted from ED records of the 12 hospital EDs that cover the five Health and Social Care Trusts in Northern Ireland. Independently trained data registration officers collect the data in accordance with the standardized criteria. Identifying cases of SH and suicide ideation involve a combination of manually checking consecutive presentations to the EDs, selecting potential cases based on keyword searches and triage coding by hospital staff. Acts of ideation include patients who have experienced thoughts of SH and/or suicide, where no physical act of harm has taken place. Where an act of SH has taken place, are not included in this study. Ideation is only recorded as a binary variable with no further information on character, duration or intensity.

Data items included age at presentation, gender and usual residential address. The relationship between alcohol and suicidal behaviour is well established (Ness *et al*., 2015), and it is recognized that intoxication can cause difficulties in treatment and management (Griffin *et al*., 2018). Alcohol involvement at the time of presentation is ascertained from ED case notes or toxicology reports. The patient’s Health and Care Number (HCN), a unique 10-digit number used throughout the Health Service in NI, is also captured enabling identification of repeat presenters, irrespective of ED of presentation, and linkage to other health services records. The successful identification of ideation presentations and the level of detail recorded for each episode is subject to routine quality checks of clinical records.

For this study, data were extracted for all ideation cases registered between 1 April 2012 and 31 December 2019. These data were linked using the HCN to the National Health Application and Infrastructure Services (NHAIS) system, which contains information on all patients registered with a primary care physician. NI has a universal, tax-financed, free at the point of service healthcare system with almost 100% population registration. NHAIS receives regular updates on date and cause of death from the General Register’s Office, though there are known delays in defining cause of death with cases such as death by suicide that require coronial review (NISRA, 2013). Mortality data were available until 31 December 2019, with deaths by suicide defined as ICD-10 codes, indicating intentional SH (ICD10: X60-X84) and sequalae of intentional SH (ICD10: Y87.0). Drug-related deaths were defined with ICD-10 codes (ICD-10: F11-F16, F18-F19, X40-X44, X60-X64, X85 and Y10-Y14) and alcohol-related deaths with ICD-10 codes (F10; G31.2; I42.6, K70; K73-K74; K86.0; X45; X65 and Y51). Accidental deaths were determined by ICD-10 codes (V01-X59). External deaths were defined as those against which cause of death was recorded under categories V01-Y98 of the ICD-10.

The study cohort consisted of all individuals aged 10+ years (defined on 1 April 2012 [*n* = 1,662,118]) and resident in NI between 1 April 2012 and 31 December 2019 who were registered with NHAIS. The lower age limit was chosen as ideation is infrequent at younger ages (Griffin *et al*., 2019). Individuals who did not present to ED with ideation were used as comparators. The patient’s address was used to append information about the characteristics of the area in which they lived. Urban/rural residence was based on a classification of settlements and grouped into three approximately equal groups: urban (comprising the two largest cities), intermediate or rural (settlements with less than 2,250 people) (NISRA, 2005). Area deprivation was based on the Northern Ireland Multiple Deprivation Measure Income index 2010 and divided into quintiles ranging from least deprived to most deprived (NISRA, 2010). The number of people registered at the same addresses was used to identify single-person households, a recognised risk factor for suicide.

The project was designed in collaboration with the Self-Harm Registry Steering Committee and approved by the Honest Broker Service Governance Board. Ethical approval was granted by the Research Ethics Committee (REC)—REF 19/LO/1601. Only a de-identified research dataset was made available to the named research group on network-isolated computers within a Trusted Research Environment, with additional disclosure control measures, including restrictions on output cell numbers to ensure confidentiality.

### Statistical analysis

The characteristics of those presenting with ideation were depicted using descriptive statistics, followed by binary logistic regression. Tests for interaction were employed to determine whether there was any effect modification between individual characteristics and the likelihood of presenting with ideation.

Separate Cox proportional hazards regression models were used to examine the risk of death by suicide, external causes and all-cause mortality among individuals presenting with ideation compared to their peers in the general population with follow-up until 31 December 2019. We extended these models to examine deaths by natural causes, accidental deaths, as well as alcohol and drug-related deaths. ICD-10 codes to categorize each of the outcomes are included in Supplementary Tables S2–S3). Models examining cause-specific mortality were censored at date of death (right censoring) for any causes of death to account for competing risks. Models were further stratified by alcohol involvement at the time of presentation. Incidence of all-cause mortality, of deaths due to external causes and of deaths due to suicide per 100,000 person-years were calculated and stratified by ideation status.

The analysis was then restricted to ideators alone to determine if there were any defining characteristics identifying those most at risk of death by suicide or other causes. Individuals were followed up from the date of first ideation presentation to either death or the end of the follow-up period. Incidence of all-cause mortality, death due to external causes and deaths due to suicide per 100,000 person-years were calculated and further stratified by year of follow-up to examine the relationship between mortality risk and time since presentation.

### Reporting

We produced a detailed analysis protocol prior to undertaking the analysis. We followed the Strengthening the Reporting of Observational Studies in Epidemiology (STROBE) and Reporting of Studies Conducted using Observational Routinely Collected Data (RECORD) checklists to guide transparent reporting of this cohort study.

## Results

The final cohort consisted of 1,662,118 individuals aged 10+ years. Between 1 April 2012 and 31 December 2019, there were 30,004 ideation presentations by 15,267 individuals. Incidence was highest in younger people, with 10–24 years olds accounting for 38% of individuals presenting to EDs with ideation. The odds of presenting with ideation were higher in males (OR_adj_ = 1.61, 95% CI 1.56, 1.67), individuals from more deprived backgrounds (most compared to least deprived: OR_adj_ = 2.48, 95% CI 2.35, 2.63) and those from urban areas (OR_adj_ = 2.12, 95% CI 2.03, 2.22) (Table 1). Individuals in single-person households were over twice as likely to present with ideation compared to multiple-person households (OR_adj_ = 2.29, 95% CI 2.18, 2.40).

**Table 1.** Socio-demographic characteristics of individuals in the cohort and likelihood (odds ratios and 95% confidence intervals) of presenting to the EDs with ideation

	Total population (% pop)	Number presenting with ideation (%)	Unadjusted OR (95% CI)	*P* value	Adjusted OR^a^ (95% CI)	*P* value
Sex						
Female	835,389 (50.2)	5,715 (37.4)	1.00		1.00	
Male	826,729 (49.7)	9,552 (62.6)	*1.70 (1.64−1.75)*	<0.001	*1.61 (1.56, 1.67)*	<0.001
Age (years)						
10−24	371,724 (22.4)	5,759 (37.7)	1.00		1.00	
25−34	277,284 (16.7)	2,975 (19.5)	*0.69 (0.66, 0.72)*	<0.001	*0.62 (0.59, 0.65)*	<0.001
35−44	271,315 (16.3)	2,860 (18.7)	*0.68 (0.65, 0.71)*	<0.001	*0.62 (0.59, 0.65)*	<0.001
45−54	267,253 (16.1)	2,452 (16.1)	*0.59 (0.56, 0.62)*	<0.001	*0.54 (0.51, 0.56)*	<0.001
55−64	201,027 (12.1)	874 (5.7)	*0.28 (0.26, 0.30)*	<0.001	*0.25 (0.23, 0.27)*	<0.001
65+	273,515 (16.5)	347 (2.3)	*0.08 (0.07, 0.09)*	<0.001	*0.07 (0.06, 0.08)*	<0.001
Income deprivation						
Least deprived	307,120 (18.5)	1,673 (11.0)	1.00		1.00	
Less deprived	321,693 (19.4)	2,465 (16.2)	*1.41 (1.32, 1.50)*	<0.001	*1.60 (1.50, 1.70)*	<0.001
Intermediate	329,766 (19.8)	3,035 (19.9)	*1.70 (1.60, 1.80)*	<0.001	*1.81 (1.70, 1.92)*	<0.001
More deprived	340,468 (20.5)	3,276 (21.5)	*1.77 (1.67, 1.88)*	<0.001	*2.00 (1.89, 2.13)*	<0.001
Most deprived	338,776 (20.4)	4,588 (30.1)	*2.51 (2.37, 2.65)*	<0.001	*2.48 (2.35, 2.63)*	<0.001
Missing	24,295 (1.5)	230 (1.5)	*1.74 (1.52, 2.00)*	<0.001	–	
Settlement band						
Rural	599,959 (36.1)	3,642 (23.9)	1.00		1.00	
Intermediate	695,574 (41.9)	6,981 (45.7)	*1.66 (1.59, 1.73)*	<0.001	*1.82 (1.74, 1.89)*	<0.001
Urban	342,390 (20.6)	4,414 (28.9)	*2.14 (2.05, 2.24)*	<0.001	*2.12 (2.03, 2.22)*	<0.001
Missing	24,295 (1.5)	230 (1.5)	*1.56 (1.37, 1.79)*	<0.001	*2.90 (2.51, 3.36)*	<0.001
Single-person household						
No	1,421,049 (85.5)	12,440 (81.5)	1.00		1.00	
Yes	165,945 (10.0)	2,182 (14.3)	*1.51 (1.44, 1.58)*	<0.001	*2.29 (2.18, 2.40)*	<0.001
Missing	75,124 (4.5)	645 (4.2)	0.98 (0.91, 1.06)	0.63	*1.15 (1.06, 1.25)*	0.001

a Adjusted for sex, age (years), income deprivation quintile, settlement band and single-person household.

### Mortality risk associated with ideation

A total of 117,580 cohort members died during the 7-year follow-up period, 1,067 (0.9%) of whom had presented with ideation. There were 5,921 deaths from external causes including 1,352 suicides, of which 7.9% and 12.3%, respectively, were in individuals who presented with ideation (Table 2). Suicide was responsible for 166 (15.6%) of ideator deaths, representing an incidence of 145 per 100,000 persons-years compared to 10 per 100,000 person-years for those with no record of ED-presenting ideation. The risk of suicide was greatest in the months most proximal to presentation, with approximately 36% of all suicide deaths occurring within the first 6 months following index presentation, 50% within 12 months and 63% within 18 months (Supplementary Table S1): the corresponding incidence rates per 100,000 person-years were, respectively, 818, 343 and 328. Almost a quarter of deaths observed among ideators were related to substance use, of which 24% were drug-related and 76% were alcohol-related. The proportion of accidental deaths was also greater among ideators, accounting for 6% of deaths compared to 0.7% of non-ideator deaths.

**Table 2.** Risk of death from suicide, external causes and all-causes following presentation with ideation, plus stratification of ideators according to the presence or absence of alcohol at time of presentation

		Died (% col)	Unadjusted HR (95% CI)	*P* value	Adjusted HR^a^ (95% CI)	*P* value
Self-inflicted deaths (*N* = 1,352)						
Ideation	No	1,186 (87.7)	*1.00*		*1.00*	
	Yes	166 (12.3)	*14.94 (12.70, 17.57)*	*<0.001*	*10.84 (9.18, 12.80)*	*<0.001*
	Yes, no alcohol	90 (6.7)	*14.09 (11.36, 17.44)*	*<0.001*	*10.74 (8.64, 13.35)*	*<0.001*
	Yes – alcohol involved	76 (5.6)	*16.10 (12.77, 20.30)*	*<0.001*	*10.96 (8.66, 13.86)*	*<0.001*
Drug-related death (*N* = 305)						
Ideation	No	244 (80.0)	1.00		1.00	
	Yes	61 (20.0)	*26.76 (20.21–35.42)*	<0.001	*15.17 (11.36–20.26)*	<0.001
	Yes, no alcohol	35 (11.5)	*26.61 (18.67–37.92)*	<0.001	*15.41 (10.73–22.11)*	<0.001
	Yes – alcohol involved	26 (8.5)	*26.96 (18.00–40.39)*	<0.001	*14.86 (9.85–22.42)*	<0.001
Alcohol-related death (*N* = 2,104)						
Ideation	No	1,912 (90.9)	1.00		1.00	
	Yes	192 (9.1)	*10.71 (9.23–12.42)*	<0.001	*10.57 (9.07–12.31)*	<0.001
	Yes, no alcohol	36 (1.7)	*3.49 (2.51–4.85)*	<0.001	*4.15 (2.98–5.78)*	<0.001
	Yes – alcohol involved	156 (7.4)	*20.5 (17.42–24.15)*	<0.001	*16.51 (13.97–19.53)*	<0.001
Accidental death (*N* = 904)						
Ideation	No	845 (93.5)	1.00		1.00	
	Yes	59 (6.5)	*7.46 (5.73–9.71)*	<0.001	*8.24 (6.29–10.81)*	<0.001
	Yes, no alcohol	27 (3.0)	*5.93 (4.04–8.70)*	<0.001	*6.59 (4.47–9.72)*	<0.001
	Yes – alcohol involved	32 (3.5)	*9.53 (6.69–13.56)*	<0.001	*10.46 (7.31–15.0)*	<0.001
External death (*N* = 5,921)						
Ideation	No	5,455 (92.1)	*1.00*		*1.00*	
	Yes	466 (7.9)	*9.11 (8.29, 10.02)*	*<0.001*	*10.65 (9.66, 11.74)*	*<0.001*
	Yes, no alcohol	235 (4.0)	*7.98 (7.01, 9.10)*	*<0.001*	*9.60 (8.40, 10.96)*	*<0.001*
	Yes – alcohol involved	231 (3.9)	*10.65 (9.33, 12.14)*	*<0.001*	*12.00 (10.49, 13.72)*	*<0.001*
Natural causes (*N* = 111,659)						
Ideation	No	111,058 (99.5)	1.00		1.00	
	Yes	601 (0.5)	*0.58 (0.53–0.63)*	<0.001	*1.93 (1.78–2.09)*	<0.001
	Yes, no alcohol	236 (0.2)	*0.39 (0.35–0.45)*	<0.001	*1.31 (1.15–1.49)*	<0.001
	Yes – alcohol involved	365 (0.3)	*0.83 (0.75–0.90)*	<0.001	*2.80 (2.52–3.10)*	<0.001
All-cause (*N* = 117,580)						
Ideation	No	116,513 (99.1)	1.00		*1.00*	
	Yes	1,067 (0.9)	0.98 (0.92, 1.04)	0.45	*3.01 (2.84, 3.20)*	*<0.001*
	Yes, no alcohol	471 (0.4)	0.75 (0.68, 0.82)	<0.001	*2.30 (2.10, 2.52)*	*<0.001*
	Yes – alcohol involved	596 (0.5)	1.29 (1.19, 1.39)	<0.001	*4.00 (3.69, 4.34)*	*<0.001*

a Adjusted for age (years), sex, income deprivation, settlement band and single-person household.

Data represent hazard ratios (HR) and 95% confidence intervals (CIs) from Cox proportional hazard models.

In unadjusted models (Table 2), the risk of suicide in those presenting with ideation was almost 15 times higher than for those with no record of ED-presenting ideation (hazard ratio [HR] = 14.94, 95% CI 12.70, 17.57), though with further adjustment for socio-demographic characteristics, this was reduced to a tenfold difference (HR_adj_ = 10.84, 95% CI 9.18, 12.80). Individuals who presented with ideation also had an elevated risk of death due to external causes (HR_adj_ = 10.65, 95% CI 9.66, 11.74). However, the greatest magnitude of risk was this risk increased for those with drug-related deaths and alcohol-related deaths (HR_adj_ = 15.17, 95% CI 11.36, 20.26 and HR_adj_ = 10.57, 95% CI 9.07, 12.31, respectively) relative to non-ideators. Table 2 shows that with the exception of death by suicide, the excess risk of death from all other mortality outcomes was modified by the presence of alcohol at the time of index presentation. For external, accidental and all-cause mortality, the risk of death was higher for those also presenting with alcohol, with an almost twofold difference in excess all-cause mortality risk (HR_adj_ = 4.00, 95% CI 3.69, 4.34 and 2.30, 95% CI 2.10, 2.52, respectively). As expected, the risk of alcohol-related death was significantly greater among ideators who presented with alcohol in their system (HR_adj_ = 16.51, 95% CI 13.97, 19.53), yet interestingly risk estimates for presentations without alcohol were also significant (HR_adj_ = 4.15, 95% CI 2.98, 5.78). The observed disparities in risk estimates for deaths due to natural causes and drug-related causes with and without alcohol involvement were comparatively less notable.

### Risk of death within those presenting with ideation

Table 3 shows that there were few socio-demographic characteristics associated with an increased risk of suicide amongst those presenting with ideation. Although suicide risk was lowest in the youngest age group (10–24 years) and highest at the oldest ages (HR_adj_ = 2.22, 95% CI 1.01–4.90 for those aged 65 years and over), there was no evidence of an age gradient. Male ideators, however, were twice as likely to die by suicide as their female peers (HR_adj_ = 2.15, 95% CI 1.49, 3.11). There was no relationship between mortality risk and either alcohol at presentation, household composition or the urban/rural nature of residence. There was a suggestion of a lower risk of suicide in ideators residing in more disadvantaged areas, though the confidence intervals were wide and crossed unity (HR_adj_ = 0.63, 95% CI 0.38, 1.06 for most compared to least deprived quintile). Those who had presented more than once with ideation had a slightly lower, though non-significant, risk of suicide (HR_adj_ = 0.83, 95% CI 0.59, 1.15).

**Table 3.** Factors associated with suicide, death by external causes and all-cause mortality following presentation to ED with suicidal ideation

	All-cause mortality (*N* = 1,067)				External death (*N* = 466)				Self-inflicted death (*N* = 166)			
	Unadjusted HR (95% CI)	*P* value	Adjusted HR^a^ (95% CI)	*P* value	Unadjusted HR (95% CI)	*P* value	Adjusted HR^a^ (95% CI)	*P* value	Unadjusted HR (95% CI)	*P* value	Adjusted HR^a^ (95% CI)	*P* value
Sex												
Female	1.00		1.00		1.00		1.00		1.00		1.00	
Male	*1.58 (1.39, 1.81)*	*<0.001*	*1.65 (1.44, 1.88)*	*<0.001*	*1.97 (1.59, 2.44)*	*<0.001*	*1.90 (1.53, 2.36)*	*<0.001*	*2.12 (1.47, 3.05)*	*<0.001*	*2.15 (1.49, 3.11)*	*<0.001*
Age at presentation (years)												
10−24	1.00		1.00		1.00		1.00		1.00		1.00	
25−34	*1.93 (1.54, 2.42)*	*<0.001*	*1.79 (1.43, 2.25)*	*<0.001*	*1.82 (1.41, 2.34)*	*<0.001*	*1.71 (1.32, 2.21)*	*<0.001*	*1.90 (1.21, 3.01)*	*0.01*	*1.83 (1.15, 2.90)*	*0.01*
35−44	*2.36 (1.89, 2.95)*	*<0.001*	*2.18 (1.74, 2.73)*	*<0.001*	1.32 (0.99, 1.75)	0.06	1.26 (0.95, 1.68)	0.11	1.62 (0.99, 2.64)	0.05	1.60 (0.98, 2.63)	0.06
45−54	*3.20 (2.59, 3.95)*	*<0.001*	*2.94 (2.37, 3.64)*	*<0.001*	1.26 (0.95, 1.68)	0.11	1.22 (0.91, 1.64)	0.18	1.58 (0.97, 2.58)	0.07	1.60 (0.96, 2.64)	0.07
55−64	*4.84 (3.84, 6.10)*	*<0.001*	*4.42 (3.48, 5.61)*	*<0.001*	1.14 (0.77, 1.70)	0.51	1.12 (0.74, 1.68)	0.59	1.73 (0.93, 3.20)	0.08	1.74 (0.92, 3.27)	0.09
65+	*8.51 (6.61, 10.97)*	*<.001*	*8.37 (6.46−10.86)*	*<.001*	1.25 (0.71, 2.18)	0.44	1.29 (0.73, 2.27)	0.38	*2.24 (1.03, 4.87)*	*0.04*	*2.22 (1.01, 4.90)*	*0.05*
Number of ideation presentations												
One	1.00		1.00		1.00		1.00		1.00		1.00	
Two+	*1.15 (1.02, 1.30)*	*0.03*	*1.12 (0.99, 1.27)*	*0.08*	*1.17 (0.97, 1.41)*	*0.08*	1.12 (0.93, 1.35)	0.24	0.85 (0.61, 1.18)	0.33	0.83 (0.59, 1.15)	0.26
Alcohol involved												
No	1.00		1.00		1.00		1.00		1.00		1.00	
Yes	*1.51 (1.34, 1.70)*	*<0.001*	*1.32 (1.17, 1.50)*	*<0.001*	*1.19 (0.99, 1.43)*	*0.06*	1.15 (0.96, 1.39)	0.13	1.05 (0.77, 1.42)	0.77	1.01 (0.74, 1.37)	0.65
Single-person household												
No	1.00		1.00		1.00		1.00		1.00		1.00	
Yes	*1.83 (1.59, 2.10)*	*<0.001*	*1.23 (1.06, 1.42)*	*0.01*	1.18 (0.93, 1.50)	0.17	1.11 (0.86, 1.42	0.43	1.23 (0.83, 1.83)	0.31	1.10 (0.73, 1.67)	0.65
Missing	*1.42 (1.09, 1.86)*	*0.01*	1.21 (0.92, 1.58)	0.18	1.22 (0.81, 1.85)	0.34	1.17 (0.77, 1.79)	0.47	1.16 (0.57, 2.37)	0.68	1.15 (0.55, 2.41)	0.70
Area deprivation quintile												
Least deprived	1.00		1.00		1.00		1.00		1.00		1.00	
Second	0.99 (0.79, 1.26)	0.97	1.03 (0.82, 1.31)	0.78	1.24 (0.86, 1.79)	0.25	1.25 (0.86, 1.80)	0.25	1.00 (0.59, 1.69)	0.99	1.02 (0.60, 1.73)	0.94
Third	0.93 (0.74, 1.17)	0.52	0.93 (0.74, 1.17)	0.54	1.07 (0.75, 1.55)	0.70	1.03 (0.72, 1.49)	0.86	0.69 (0.40, 1.19)	0.18	0.68 (0.39, 1.17)	0.16
Fourth	1.00 (0.80, 1.25)	0.99	1.03 (0.82, 1.29)	0.79	1.23 (0.86, 1.75)	0.26	1.20 (0.84, 1.71)	0.34	0.70 (0.41, 1.19)	0.19	0.71 (0.41, 1.21)	0.21
Most deprived	0.95 (0.77, 1.18)	0.64	0.91 (0.74, 1.13)	0.41	1.08 (0.76, 1.52)	0.68	1.00 (0.71, 1.42)	0.98	0.63 (0.38, 1.05)	0.08	0.63 (0.38, 1.06)	0.08
Missing	1.04 (0.62–1.73)	0.88	–		1.47 (0.72−3.03)	0.29	–		0.93 (0.28, 3.09)	0.90	–	
Settlement band												
Rural	1.00		1.00		1.00		1.00		1.00		1.00	
Intermediate	1.14 (0.98, 1.34)	0.10	*1.19 (1.01, 1.40)*	*0.01*	1.21 (0.95, 1.55)	0.11	1.22 (0.95, 1.57)	0.11	1.33 (0.89, 1.99)	0.16	1.36 (0.91, 2.05)	0.80
Urban	1.16 (0.98, 1.37)	0.09	*1.25 (1.05, 1.48)*	*0.03*	1.24 (0.95, 1.61)	0.12	1.23 (0.94, 1.61)	0.13	1.02 (0.65, 1.61)	0.93	1.06 (0.67, 1.69)	0.13
Missing	1.20 (0.73, 1.96)	0.48	1.08 (0.63, 1.84)	0.78	1.53 (0.77, 3.04)	0.22	1.55 (0.72, 3.31)	0.29	1.42 (0.44, 4.63)	0.56	1.04 (0.29, 3.73)	0.95

a Adjusted for sex, age at presentation (years), income deprivation, settlement band, single-person household, number of ideation presentations and alcohol involvement.

Data represent hazards ratios (95% CIs) from Cox proportional hazards models.

The pattern for all-cause mortality differed. The eightfold gradient across the age spectrum was much steeper, though the male excess (HR_adj_ = 1.65, 95% CI 1.44, 1.88) was more muted than for suicide risk. A higher risk was now evident amongst those in single-person house (HR_adj_ = 1.42, 95% CI 1.09, 1.86) and amongst ideators with significant alcohol intake at presentation (HR_adj_ = 1.51, 95% CI 1.34, 1.70). There was no relationship between all-cause mortality and deprivation though risk was moderately higher for those in larger conurbations (e.g., HR_adj_ = 1.25, 95% CI 1.05, 1.48 for urban compared to rural dwellers). People who presented with ideation on two or more occasions were also slightly more likely to die from any cause than their peers who presented only once (HR_adj_ = 1.15, 95% CI 1.02, 1.30). The patterns observed in models examining the risk of alcohol-related death were similar with and without adjustment for alcohol involvement at the time of presentation (Supplementary Table S4). Risk of alcohol-related death was significantly higher in those with multiple ideation presentations (HR_adj_ = 1.37, 95% CI 1.03, 1.83), those presenting with alcohol involvement (HR_adj_ = 3.78, 95% CI 2.62, 5.45) and those with increasing age. Male gender was associated with a 38% increased risk with adjustment for socio-demographic characteristics (HR_adj_ = 1.38, 95% CI 1.01, 1.87); however, this was attenuated and lost significance with adjustment for alcohol involvement (HR_adj_ = 1.32, 95% CI 0.97, 1.79). Likewise, there was no significant relationship between mortality risk and either settlement band, household composition or area-level deprivation, with the exception of those living in the most deprived quintile ((HR_adj_ = 0.60, 95% CI 0.37, 0.95). The socio-demographic and area-level pattern for deaths by natural causes (available on request) and deaths by external causes, which also subsumes self-inflicted deaths, lies between the pattern for suicide and that for all-cause mortality risk. We did not identify any statistically significant risk factors for death by drug-related or accidental causes following ideation (available on request), although given the small number of deaths in these categories, it is plausible that this resulted from insufficient statistical power.

## Discussion

This study reaffirms that ideation presentations to the ED are a common phenomenon, with an incidence half that of SH (Griffin *et al*., 2019) and exhibiting similar demographic and socio-economic characteristics (Corcoran *et al*., 2007), though this is probably because ideation often precedes suicidal behaviours such as SH (Klonsky *et al*., 2016). Individuals from multiple-person households were half as likely to present with ideation as those living alone underpinning the protection that co-habitation may offer against SH and suicide (Shaw *et al*., 2021).

Patients presenting with ideation were 10 times more likely to die from suicide than their peers (HR 10.84, 95% CI 9.18, 12.80), accounting for more than one-in-ten of all suicide deaths; half of those deaths occurred in the 12 months following ED presentation. Alcohol intake at presentation did not moderate this risk. Our findings are consistent with a recent study (Goldman-Mellor *et al*., 2019) that reported a 1-year post-ED presentation suicide incidence rate of 384.5/100,000 for individuals presenting to ED with ideation, which is similar to the 343/100,000 reported in the current study. The threefold increased risk for ideators reported in the recent meta-analysis is lower (McHugh *et al*., 2019), perhaps because it was based largely on ideation derived from primary care records or from self-reported structured interviews and thus captures a wide spectrum of individuals experiencing suicidal thoughts (Geulayov *et al*., 2019; McHugh *et al*., 2019). It is likely that individuals presenting to hospital are experiencing episodes of ideation that are more significant in terms of intensity, duration or associated distress and may be associated with greater suicidal intent. This may account for the relative proximity to death in the current study, which is contrasting to other studies such as Berman (2018) who suggest that SI is a better indicator of future rather than proximate risk.

There was, however, little that differentiated ideation presenters who were more likely to take their lives. Suicide risk was twice as likely in males and slightly, though non-significantly, raised amongst those living in more affluent areas. Similar associations have been noted for those presenting with SH (Berman, 2018) but, and again as with SH (Chan *et al*., 2016; Steeg *et al*., 2018), collectively these characteristics are not sufficiently strong or specific to facilitate targeted intervention. This is in marked contrast to the strong association between demographic and socio-economic factors and likelihood of ideation presentation and underscores the disconnection of factors associated with ideation and those associated with transition to suicidal behaviours (Klonsky *et al*., 2016) and the increased need for more studies of truly proximal factors (Berman, 2018).

Ideators also had a 10- to 11-fold increased risk of death from external causes. This subsumes the 166 suicide deaths but also includes almost twice as many (*n* = 300) deaths from other causes, including accidental poisoning, events of undetermined intent, falls, other accidents and deaths due to assault and violence. All-cause mortality risk was also three times higher than expected. Increased mortality from external and natural causes are also recognized sequelae of SH, leading to an estimated 10–30 years of life lost (Bergen *et al*., 2012). The causes are probably multifactorial but may be attributable to other concurrent psychiatric disorders (Hawton *et al*., 2013) and poorly treated physical health problems (Thornicroft, 2011). Reverse causation is also possible, that is, that chronic disease per se may increase the risk of both ideation and suicidal behaviours. Risky behaviours such as alcohol and drugs are also associated with SH and ideation (Griffin *et al*., 2018). Indeed, Bergen *et al*. (2014) found that individuals presenting to EDs with both SH and alcohol involvement were more likely to die from alcohol-related causes. This accords with the higher excess mortality from external and all-causes in ideators presenting with significant alcohol intake in the current study. Indeed, we observed that death resulting from substance misuse, particularly death from alcohol-related causes, is a significant and prevalent cause of death among individuals who present with ideation, accounting for just under a quarter of all deaths observed. Notably, the risk estimate for alcohol-related deaths among ideators was commensurate with that of suicide risk and this remained significant even when adjustment was made for alcohol intake at presentation. However, death from drug-related causes carried the single greatest cause-specific risk of all outcomes examined in the current study, suggesting that whilst suicide prevention may be the primary focus of interventions aimed at those experiencing ideation, death from other preventable causes should also be considered.

To our knowledge this is the first population-wide study to examine the mortality risk of those presenting to EDs with ideation using data derived from a dedicated registry with validated case ascertainment methods. This circumvents many of the limitations of previous survey-based studies which are subject to responder and recall biases and small sample sizes owing to the rarity of suicide. There are, however, some limitations to consider. The registry only records ideation as a binary, and information about the severity or duration of ideation or the extent of any associated suicidal planning that might provide better identification of those most at risk is absent. The study was based on ED-presenting ideation and therefore of limited generalizability to individuals in the community who experience thoughts of SH or suicide. It was also not possible in the current study to examine the influence of known pre-existing psychiatric disorders, though the Californian study showed that psychiatric morbidity recorded at the ED was not strongly associated with suicide risk in those presenting to EDs with SI (Goldman-Mellor *et al*., 2019).

In conclusion, this study confirms that presentations to EDs with SI is an important if neglected public health issue. The increased risk of death in the period following ED presentation suggests that EDs are a critical setting for mental health assessments and a catalyst for community-based care. Previous analysis of SH data (Arensman *et al*., 2018) shows that most patients are discharged home following assessment, with only 8% admitted to a psychiatric ward; therefore, the risks of subsequent presentation to the ED with SH are high (Arensman *et al*., 2018). However, there are no evidence-based clinical guidelines for ideation presentations as there are for SH (National Institute for Health and Clinical Excellence, 2004; National Institute for Health and Clinical Excellence, 2022), and it is not known if ideation, in the absence of suicide attempts or actual SH, stimulates the same community response, such as access to mental health teams or talking therapies, as SH will.

## Data Availability

The data used in this study are available in the Honest Broker Service (HBS) within the Business Services Organisation (BSO), Northern Ireland, but as restrictions apply, they are not publicly available. All proposals to use data are subject to review by an independent HBS Governance Board (HBSGB). Before any data can be accessed, approval must be given by the HBSGB. When access has been granted, it is gained through a privacy protecting safe haven and remote access system. HBS has established an application process to be followed by anyone who would like to access data, which can be found at https://hscbusiness.hscni.net/services/2454.htm.

## Supplementary material

For supplementary material accompanying this paper visit http://doi.org/10.1017/S2045796023000203. click here to view supplementary material

## References

[ref1] Arensman E, Griffin E, Daly C, Corcoran P, Cassidy E and Perry IJ (2018) Recommended next care following hospital-treated self-harm: Patterns and trends over time. *PLoS One* 13, e0193587. 10.1371/journal.pone.0193587 PMC5832269 29494659

[ref2] Bergen H, Hawton K, Waters K, Ness J, Cooper J, Steeg S and Kapur N (2012) Premature death after self-harm: A multicentre cohort study. *The Lancet* 380, 1568–1574. 10.1016/S0140-6736(12)61141-6 22995670

[ref3] Bergen H, Hawton K, Webb R, Cooper J, Steeg S, Haigh M, Ness J, Waters K and Kapur N (2014) Alcohol-related mortality following self-harm: A multicentre cohort study. *JRSM Open* 5(8), 2054270414533326. 10.1177/2054270414533326 PMC4100241 25289146

[ref4] Berman AL (2018) Risk factors proximate to suicide and suicide risk assessment in the context of denied suicide ideation. *Suicide Life Threatening Behaviours* 48, 340–352. 10.1111/sltb.12351 28429385

[ref5] Carr MJ, Ashcroft DM, Kontopantelis E, While D, Awenat Y, Cooper J, Chew-Graham C, Kapur N and Webb RT (2016) Clinical management following self-harm in a UK-wide primary care cohort. *Journal of Affective Disorders* 197, 182–188. 26994436 10.1016/j.jad.2016.03.013 PMC4870375

[ref6] Chan MK, Bhatti H, Meader N, Stockton S, Evans J, O’Connor RC, Kapur N and Kendall T (2016) Predicting suicide following self-harm: Systematic review of risk factors and risk scales. *The British Journal of Psychiatry* 209(4), 277–283. 27340111 10.1192/bjp.bp.115.170050

[ref7] Corcoran P, Arensman E and Perry IJ (2007) The area-level association between hospital-treated deliberate self-harm, deprivation and social fragmentation in Ireland. *Journal of Epidemiology and Community Health* 61, 1050–1055. 18000126 10.1136/jech.2006.055855 PMC2465671

[ref8] Geulayov G, Casey D, Bale L, Brand F, Clements C, Farooq B, Kapur N, Ness J, Waters K, Tsiachristas A and Hawton K (2019) Suicide following presentation to hospital for non-fatal self-harm in the Multicentre Study of Self-harm: A long-term follow-up study. *The Lancet Psychiatry* 6(12), 1021–1030. 31706930 10.1016/S2215-0366(19)30402-X

[ref9] Goldman-Mellor S, Olfson M, Lidon-Moyano C and Schoenbaum M (2019) Association of suicide and other mortality with emergency department presentation. *JAMA Network Open* 2, e1917571. 10.1001/jamanetworkopen.2019.17571 PMC6991205 31834399

[ref10] Goodfellow B, Kõlves K and de Leo D (2019) Contemporary definitions of suicidal behavior: A systematic literature review. *Suicide & Life-Threatening Behavior* 49, 488–504. 29574910 10.1111/sltb.12457

[ref11] Griffin E, Arensman E, Perry IJ, Bonner B, O’Hagan D, Daly C and Corcoran P (2018) The involvement of alcohol in hospital-treated self-harm and associated factors: Findings from two national registries. *Journal of Public Health* 40, e157–e163. 28498968 10.1093/pubmed/fdx049

[ref12] Griffin E, Bonner B, O’Hagan D, Kavalidou K and Corcoran P (2019) Hospital-presenting self-harm and ideation: Comparison of incidence, profile and risk of repetition. *General Hospital Psychiatry* 61, 76–81. 31731175 10.1016/j.genhosppsych.2019.10.009

[ref13] Griffin E, Kavalidou K, Bonner B, O’Hagan D and Corcoran P (2020) Risk of repetition and subsequent self-harm following presentation to hospital with suicidal ideation: A longitudinal registry study. *EClinicalMedicine* 23, 100378. 10.1016/j.eclinm.2020.100378 PMC7280762 32529177

[ref14] Harmer B, Lee S, Duong TVH and Saadabadi A (2023) Suicidal ideation. In *StatPearls*. Treasure Island, FL: StatPearls Publishing. 33351435

[ref15] Hawton K, Saunders K, Topiwala A and Haw C (2013) Psychiatric disorders in patients presenting to hospital following self-harm: A systematic review. *Journal of Affective Disorders* 151, 821–830. 24091302 10.1016/j.jad.2013.08.020

[ref16] Jobes DA and Joiner TE (2019) Reflections on suicidal ideation. *Crisis* 40, 227–230. 31274031 10.1027/0227-5910/a000615

[ref17] Klonsky ED, May AM and Saffer BY (2016) Suicide, suicide attempts, and suicidal ideation. *Annual Review of Clinical Psychology* 12, 307–330. 10.1146/annurev-clinpsy-021815-093204 26772209

[ref18] McHugh CM, Corderoy A, Ryan CJ, Hickie IB and Large MM (2019) Association between suicidal ideation and suicide: Meta-analyses of odds ratios, sensitivity, specificity and positive predictive value. *BJPsych Open* 5, e18. 10.1192/bjo.2018.88 PMC6401538 30702058

[ref19] National Institute for Health and Clinical Excellence (2004) *Self-Harm: The Short-Term Physical and Psychological Management and Secondary Prevention of Self-Harm in Primary and Secondary Care*. Leicester: British Psychological Society. 21834185

[ref20] National Institute for Health and Clinical Excellence (2022) Self-harm: Assessment, management and preventing recurrence https://www.nice.org.uk/guidance/ng225/resources/selfharm-assessment-management-and-preventing-recurrence-pdf-66143837346757. 36595613

[ref21] Ness J, Hawton K, Bergen H, Cooper J, Steeg S, Kapur N, Clarke M. and Waters K (2015) Alcohol use and misuse, self-harm and subsequent mortality: An epidemiological and longitudinal study from the multicentre study of self-harm in England. *Emergency Medicine Journal* 32(10), 793–799. 25564479 10.1136/emermed-2013-202753

[ref22] Nock MK, Borges G, Bromet EJ, Alonso J, Angermeyer M, Beautrais A, Bruffaerts R, Chiu WT, De Girolamo G, Gluzman S and De Graaf R (2008) Cross-national prevalence and risk factors for suicidal ideation, plans and attempts. *The British Journal of Psychiatry* 192(2), 98–105. 18245022 10.1192/bjp.bp.107.040113 PMC2259024

[ref23] Northern Ireland Statistics and Research Agency (2005). Report of the inter-departmental urban-rural definition group: Statistical classification and delineation of settlements. https://www.nisra.gov.uk/sites/nisra.gov.uk/files/publications/ur_main.pdf (accessed13 June 2022).

[ref24] Northern Ireland Statistics and Research Agency (2010) Northern Ireland multiple deprivation *Measure 2010*. https://www.nisra.gov.uk/sites/nisra.gov.uk/files/publications/NIMDM_2010_Report_0.pdf (accessed 13 June 2022).

[ref25] Northern Ireland Statistics and Research Agency (2013) Suicide Statistics in Northern Ireland: Impact of time taken to investigate the death. http://www.nisra.gov.uk/demography/default.asp31.htm (accessed 13 June 2022).

[ref26] Public Health Agency (2019) Northern Ireland Registry of Self-Harm: Annual Report 2017/18. https://www.publichealth.hscni.net/sites/default/files/2019-07/NIRSHAnnualReport2017.18finalforpublication_0.pdf (accessed 13 June 2022).

[ref27] Shaw RJ, Cullen B, Graham N, Lyall DM, Mackay D, Okolie C, Pearsall R, Ward J, John A and Smith DJ (2021) Living alone, loneliness and lack of emotional support as predictors of suicide and self-harm: A nine-year follow up of the UK Biobank cohort. *Journal of Affective Disorders* 279, 316–323. 33096330 10.1016/j.jad.2020.10.026 PMC7758739

[ref28] Steeg S, Quinlivan L, Nowland R, Carroll R, Casey D, Clements C, Cooper J, Davies L, Knipe D, Ness J and O’Connor RC (2018) Accuracy of risk scales for predicting repeat self-harm and suicide: A multicentre, population-level cohort study using routine clinical data. *BMC Psychiatry* 18, 113. 10.1186/s12888-018-1693-z PMC5921289 29699523

[ref29] Stene-Larsen K and Reneflot A (2019) Contact with primary and mental health care prior to suicide: A systematic review of the literature from 2000 to 2017. *Scandinavian Journal of Public Health* 47, 9–17. 29207932 10.1177/1403494817746274

[ref30] Thornicroft G (2011) Physical health disparities and mental illness: The scandal of premature mortality. *British Journal of Psychiatry* 199, 441–442. 10.1192/bjp.bp.111.092718 22130744

