# Supplementary material for: Emergency department presentations with suicide and self-harm ideation: a missed opportunity for intervention?
Source: Epidemiol Psychiatr Sci. 2023 Apr 18;32:e24. doi: 10.1017/S2045796023000203 (PMC10130835; doi:10.1017/S2045796023000203)
Supplement: Supplementary file 1 [file S2045796023000203sup001.docx]

**Supplementary Table 1. Incidence of all-cause mortality, death by external causes and death due to self-inflicted injuries by number of months since first recorded ideation presentation per 100,000 person years (95% confidence intervals)**

|  | All-cause mortality (N=1,067) | | External death (N=466) | | Self-inflicted death (N=166) | |
| --- | --- | --- | --- | --- | --- | --- |
|  | Number of deaths (% col) | Incidence rate | Number of deaths (% col) | Incidence rate | Number of deaths (% col) | Incidence rate |
| 6 months | 159 (14.9) | 2168 | 98 (21.0) | 1336 | 60 (36.1) | 818 |
| 12 months | 123 (11.5) | 1838 | 64 (13.7) | 957 | 23 (13.9) | 344 |
| 18 months | 108 (10.1) | 1770 | 53 (11.4) | 869 | 20 (12.0) | 328 |
| 24 months *(<18 months for self-inflicted) | 89 (8.3) | 1616 | 38 (8.2) | 690 | 63 (38.0) | 179 |
| >24 months | 588 (55.1) | 1975 | 213 (45.7) | 715 | - | - |

*Incidence rates for self-inflicted deaths cut off at >18 months to ensure conformity with principles of statistical disclosure control

**Supplementary Table 2. ICD-10 codes identifying external causes of morbidity and mortality (V01-Y98)**

| **ICD-10 Code** | **Description** |
| --- | --- |
|  | |
| **V01-X59** | **Accidents** |
| V01-V99 | Transport Accidents |
| W00-W19 | Falls |
| W20-W49 | Exposure to inanimate mechanical forces |
| W50-W64 | Exposure to animate mechanical forces |
| W65-W74 | Accidental drowning and submersion |
| W75-W84 | Other accidental threats to breathing |
| W85-W99 | Exposure to electric current, radiation and extreme ambient air temperature and pressure |
| X00-X09 | Exposure to smoke, fire and flames |
| X10-X19 | Contact with heat and hot substances |
| X20-X29 | Contact with venomous animals and plants |
| X30-X39 | Exposure to forces of nature |
| X40-X49 | Accidental poisoning by and exposure to noxious substances |
| X50-X57 | Overexertion, travel and privation |
| X58-X59 | Accidental exposure to other and unspecified factors |
|  | |
| **X60-X84** | **Intentional self-harm** |
| X60-X69 | Intentional self-harm by poisoning |
| X70 | Intentional self-harm by hanging, strangulation and suffocation |
| X71 | Intentional self-harm by drowning and submersion |
| X72 | Intentional self-harm by handgun discharge |
| X73 | Intentional self-harm by rifle, shotgun and larger firearm discharge |
| X74 | Intentional self-harm by other and unspecified firearm discharge |
| X75 | Intentional self-harm by explosive material |
| X76 | Intentional self-harm by smoke, fire and flames |
| X77 | Intentional self-harm by steam, hot vapours and hot objects |
| X78 | Intentional self-harm by sharp object |
| X79 | Intentional self-harm by blunt object |
| X80 | Intentional self-harm by jumping from a high place |
| X81 | Intentional self-harm by jumping or lying before moving object |
| X82 | Intentional self-harm by crashing of motor vehicle |
| X83 | Intentional self-harm by other specified means |
| X84 | Intentional self-harm by unspecified means |
|  | |
| **X85 – Y09** | **Assault** |
|  | |
| **Y10-34** | **Events of undetermined intent** |
| Y10-Y19 | Poisoning, undetermined intent |
| Y20 | Hanging, strangulation and suffocation, undetermined intent |
| Y21 | Drowning and submersion, undetermined intent |
| Y22 | Handgun discharge, undetermined intent |
| Y23 | Rifle, shotgun and larger firearm discharge, undetermined intent |
| Y24 | Other and unspecified firearm discharge, undetermined intent |
| Y25 | Contact with explosive material, undetermined intent |
| Y26 | Exposure to smoke, fire and flames, undetermined intent |
| Y27 | Contact with steam, hot vapours and hot objects, undetermined intent |
| Y28 | Contact with sharp object, undetermined intent |
| Y29 | Contact with blunt object, undetermined intent |
| Y30 | Falling, jumping or pushed from a high place, undetermined intent |
| Y31 | Falling, lying or running before or into moving object, undetermined intent |
| Y32 | Crashing of motor vehicle, undetermined intent |
| Y33 | Other specified events, undetermined intent |
| Y34 | Unspecified event, undetermined intent |
|  |  |
| Y35-Y36 | Legal intervention and operations of war |
|  | |
| **Y40-Y84** | **Complications of medical and surgical care** |
|  | |
| **Y85-Y89** | **Sequalae of external causes of morbidity and mortality** |
| Y85 | Sequelae of transport accidents |
| Y86 | Sequelae of other accidents |
| Y87 | Sequelae of intentional self-harm, assault and events of undetermined intent |
| Y87.0 | Sequelae of intentional self-harm |
| Y87.1 | Sequelae of assault |
| Y87.2 | Sequelae of events of undetermined intent |
| Y88 | Sequelae with surgical and medical care as external cause |
| Y89 | Sequalae of other external causes |
|  | |
| **Y90-Y98** | **Supplementary factors related to causes of morbidity and mortality classified elsewhere** |
|  | |

Code derived from International Statistical Classification of Diseases and Related Health Problems 10th Revision (ICD-10)

Suicide deaths were identified using codes X60-X84, and Y87.0

Accidental deaths were identified using codes V01-X59

**Supplementary Table 3. ICD-10 codes identifying alcohol and drug-related causes of death**

| **ICD-10 Code** | **Description** |
| --- | --- |
|  | |
| **Alcohol-related causes of death** | |
| F10 | Mental and behavioral disorders due to use of alcohol |
| G31.2 | Degeneration of the nervous system due to alcohol |
| G62.1 | Alcoholic polyneuropathy |
| I42.6 | Alcohol gastritis |
| K70 | Alcoholic liver disease |
| K73 | Chronic hepatitis, not elsewhere classified |
| K74 | Fibrosis and cirrhosis of liver (excluding K74.3-K74.5 - Biliary cirrhosis) |
| K86.0 | Alcohol-induced chronic pancreatitis |
| X45 | Accidental poisoning by and exposure to alcohoL |
| X65 | Intentional self-poisoning by and exposure to alcohol |
| Y15 | Poisoning by and exposure to alcohol - undetermined intent |
|  | |
| **Drug-related causes of death** | |
| F11-F16, F18-F19 | Mental and behavioral disorders due to drug use |
| X40-X44 | Accidental self-poisoning by drugs, medicaments and biological substances |
| X60-X64 | Intentional self-poisoning by drugs, medicaments and biological substances |
| X85 | Assault by drugs, medicaments and biological substances |
| Y10-Y14 | Poisoning by drugs, medicaments and biological substances - undetermined intent |
|  | |
